# Complete genome sequence of marine prosthecate bacterium *Fretibacter rubidus* type strain JCM 15585

**DOI:** 10.1128/mra.00986-24

**Published:** 2024-10-14

**Authors:** Breah LaSarre, Mark L. Watson, Emma M. Corby, Amelia M. Randich

**Affiliations:** 1 Department of Plant Pathology, Entomology, and Microbiology, Iowa State University, Ames, Iowa, USA; 2 Department of Biology, University of Scranton, Scranton, Pennsylvania, USA; Portland State University, Portland, Oregon, USA

**Keywords:** Alphaproteobacteria, Caulobacterales, *Robigintomaculaceae*, prosthecate, dimorphic bacteria

## Abstract

*Fretibacter rubidus* is a dimorphic, prosthecate member of the family *Maricaulaceae*, order Caulobacterales, in the class Alphaproteobacteria. Here, we report the genome of type strain JCM 15585^T^ (JC2236^T^), sequenced with Oxford Nanopore Technology.

## ANNOUNCEMENT

*Fretibacter rubidus* was isolated from seawater off Jeju Island, Republic of Korea in 2013 and was characterized as a prosthecate, budding bacterium of the family *Hyphomonadaceae* (1). It is the only member of its genus and has highest 16S rRNA similarity to *Hellea balneolensis* 26III/A02/215T (96.17%) (1). The *Fretibacter* genus has since been moved to the family *Robigintomaculaceae*, along with the genera *Robiginitomacilum*, *Hellea*, *Litorimonas*, and *Algimonas* (2). Members of this family of marine caulobacters display a cryptic mixture of reproductive strategies, with some species reported as reproducing by binary fission and others by budding. Sequencing the *F. rubidus* genome will allow for further phylogenetic and bioinformatic analysis of this family and greater understanding of reproductive plasticity in the Caulobacterales order.

Strain JCM 15585 was grown in Marine Broth (2216, BD Difco, Sigma) at 30°C, 220 rpm, until mid-exponential (OD_600_ 1.27) from a single colony from a lab dimethylsulfoxide (DMSO) stock (propagated from lyophilisate from JCM). Cells were washed in phosphate-buffered saline (PBS) and resuspended in DNA/RNA Shield (Zymo Research) prior to DNA isolation, library preparation, and sequencing performed at Plasmidsaurus. Total genomic DNA was isolated using a Quick-DNA Miniprep Plus Kit (Zymo). Libraries were prepared with the Oxford Nanopore Technologies (ONTs) Rapid Barcoding Kit 96 V14 and sequenced on a PromethION P24 with R10.4.1. flow cell. Base calling was performed with ont-doradod-for-promethion v7.1.4 in super-accurate mode (model dna_r10.4.1_e8.2_400bps_sup@v4.2.0), with a read Q-score cutoff of 10, producing 68,771 reads (*N_50_*: 11,664 bp). Following elimination of the worst 5% of reads and reads <1,000 bp (--min_length 1,000 --keep_percent 95) using Filtlong v0.2.1 (https://github.com/rrwick/Filtlong), the remaining 45,348 reads (*N_50_* of 12,314 bp) were used as input to five assemblers: NextDenovo v2.5.2 (3), Miniasm + Minipolish v0.3-r179/0.1.3 (4, 5), Flye v2.9.3 (6), Raven v1.8.3 (7), and Canu v2.2 (8). The resulting five independent assemblies were used to generate a consensus assembly with Trycycler v0.5.4 (9) as outlined in the detailed documentation (https://github.com/rrwick/Trycycler/wiki). Following polishing with Medaka v1.11.3 (https://github.com/nanoporetech/medaka; ONT), the single contig was manually rotated to start at the *dnaA* gene as predicted by bakta v1.9.3 (10). Default parameters were used except where otherwise noted.

The JCM 15585 assembly comprises a single, circular 3,450,490 bp chromosome (51.13% GC), with average read coverage of 112× [per Quast v5.2.0 (11))], and estimated 99.99% completeness with 0.5% contamination [per CheckM2 v1.0.2 (12)], The absence of plasmids in the assembly was supported by unfruitful plasmid DNA extraction (Zyppy Miniprep kit, Zymo). The assembly was submitted to the Genbank database with concomitant annotation using the NCBI Prokaryotic Genome Annotation Pipeline, which identified 3,119 protein-coding genes, 37 tRNAs, and a single rRNA operon.

## Data Availability

The sequencing read data set [Sequence Read Archive (SRA): SRR29931580], as well as the chromosome (GenBank: CP163423) are deposited in the National Center for Biotechnology Information database under BioProject PRJNA1138649 and BioSample SAMN42730191.
